# Supplementary material for: hTERT DNA Methylation Analysis Identifies a Biomarker for Retinoic Acid-Induced hTERT Repression in Breast Cancer Cell Lines
Source: Biomedicines. 2022 Mar 17;10(3):695. doi: 10.3390/biomedicines10030695 (PMC8945736; doi:10.3390/biomedicines10030695)
Supplement: Supplementary file 1 [file biomedicines-10-00695-s001.zip › biomedicines-1628346-supplementary.pdf]

Supplementary Materials

Supplementary Table S1: Primers sequences (5′ to 3′) and amplification conditions used for sequencing and *hTERT* expression analyses. The positions of the amplicons are indicated in Supplementary Figures S3 and S4.

Primer name	Primers*	Annealing °C
	**Sequencing**	
hTERT-5kb region-seq	FW : ACCCTTCTCAAGGGAAAACCAGA	60
	RV : TGGAATCATTCAATCCTTGGGG	
hTERT-promoter-seq	FW (-712) : AACAGATTTGGGGTGGTTTG	60
	RV (+120) : AGCACCTCGCGGTAGTGG	
	**Bisulfite sequencing**	
hTERT-5kb region-BS	FW : TTTGGAGAGAGGAGTTTGAG	53
	RV : TCATTCAATCCTTAAAAATAAAATAAATA	
hTERT-promoter-BS	FW : GGGTTTGTGTTAAGGAGTTTAAGT	60
	RV : CCAACCCTAAAACCCCAAC	
	**PCR**	
hTERT-qPCR	FW : CGGAAGAGTGTCTGGAGCAA	58
	RV : CTCCCACGACGTAGTCCATG	
GAPDH-qPCR	FW : CACCCATGGCAAATTCCATGGC	58
	RV : GCATTGCTGATGATCTTGAGGCT	
PBGD-qPCR	FW : GGAATGCATGTATGCTGTGG	61
	RV : CAGGTACAGTTGCCCATCC	

Supplementary Figure S1. The DNA sequence corresponding to the 828bp region from -666 to +166 nucleotide positions from TSS. All analyzed CpG dinucleotides are in bold. The nucleotide positions of the transcription site (TTS +1) and the translation initiation codon (ATG) are indicated. The sequences of the primers used for PCR amplification in sequencing experiments are shown in italic type. Binding sites for major transcription factors such as Sp1, WT1, and Myc/Mad (E-Box) are underlined. The dotted box indicates the THOR region defined as a 433 base pair genomic region within the *hTERT*. promoter (Chr5:1,295,321–1,295,753, GRCh37/hg19). Blue letters show the C228T and C250T mutations.

***hTERT* promoter region I** Chr5: 1,294,997-1,295,824 (GRCh37/hg19)

GGGCCTGTGTCAAGGAGCCCAAGT<u>**CG**^{1}**CG**^{2}GGGA</u>AGTGTTGCAGGGAGGCACTC**CG**^{3}GGAGGTCC**CG**^{4}**CG**^{5}TGCC**CG**^{6}TCCAGGGAGCAATG
MZF2

CG^{7}TCCT**CG**^{8}GGTT**CG**^{9}<u>TCCCCAGC</u>**CG**^{10}**CG**^{11}TCTA**CG**^{12}**CG**^{13}CCTC<u>**CG**^{14}TCCTCCCCTTCA</u>**CG**^{15}TC**CG**^{16}GCATT**CG**^{17}TGGTGCC**CG**^{18}GAGCC**C**
MZF2 MZF2

G^{19}A**CG**^{20}CCC**CG**^{21}**CG**^{22}TC**CG**^{23}GACCTGGAGGCAGCCCTGGGTCTC**CG**^{24}GATCAGGCCAG**CG**^{25}GCCAAAGGGT**CG**^{26}C**CG**^{27}CA**CG**^{28}CACCT

GTTCCCAGGGCCTCCACATCATGGCCCCTCCCT**CG**^{29}GGTTACCCCACAGCCTAGGC**CG**^{30}ATT**CG**^{31}ACCTCTCTC**CG**^{32}CTGGGGCCCT**CG**^{33}CTG

G**CG**^{34}TCCCTGCACCCTGGGAG<u>**CG**^{35}**CG**^{36}AG**CG**^{37}G**CG**^{38}**CG**^{39}</u>**CG**^{40}GG**CG**^{41}GGGAAG**CG**^{42}**CG**^{43}GCCCAGACCCC**CG**^{44}GGTC**CG**^{45}CC**CG**^{46}G
WT1

AGCAGCTG**CG**^{47}CTGT**CG**^{48}GGGCCAGGC**CG**^{49}GGCTCCCAGTGGATT**CG**^{50}**CG**^{51}GGCACAGA**CG**^{52}CCCAGGAC**CG**^{53}**CG**^{54}CTTCC<u>CA**CG**^{55}TG</u>
E-Box

G**CG**^{56}GAGGGACTGGGGACC**CG**^{57}GGCACC**CG**^{58}TCCTGCCCCTTCACCTTCCAGCT<u>C**CG**^{59}CCTC</u>CTC**CG**^{60}**CG**^{61}**CG**^{62}GACC<u>C**CG**^{63}CCC</u>**CG**^{64}TC
sp1 sp1

C250T C228T
C**CG**^{65}ACCCCTCC**CG**^{66}GGTCCC**CG**^{67}G<u>CCCAGCCCC</u>CTC**CG**^{68}GGCCCT<u>CCCAGCCCCTCCC</u>CTTCCTTTC**CG**^{69}**CG**^{70}GCC<u>C**CG**^{71}CCC</u>TCTCCT**CG**
sp1 sp1 sp1

→ **TTS +1**
^{72}**CG**^{73}G**CG**^{74}**CG**^{75}AGTTTCAGGCAG**CG**^{76}CTG**CG**^{77}TCCTGCTG**CG**^{78}<u>CA**CG**^{79}TG</u>GGAAGCCCTGGCCC**CG**^{80}GCCACCCC**CG**^{81}**CG**^{82}**ATG**C**CG**^{83}
E-Box

CG^{84}**CG**^{85}CT<u>CCC**CG**^{86}CTGC**CG**^{87}AGC**CG**^{88}TG**CG**^{89}CTCCC</u>TGCTG**CG**^{90}CAGCCACTAC**CG**^{91}**CG**^{92}AGGTGCTGC**CG**^{93}CTGGCCA**CG**^{94}TT**CG**^{95}T
CTCF

G**CG**^{96}G**CG**^{97}C*CTGGGGCCCCAGGGCTGG*

Supplementary Figure S2. DNA sequence corresponding to the 696bp region from -5600 to -4904 nucleotide positions from TSS. All analyzed CpG dinucleotides are in bold. The sequences of the primers used for PCR amplification in sequencing experiments are shown in italic type. Putative binding sites for Sp1, GABPA, and RAR-RXR transcription factors are underlined. CpG^{8} (chr5: 1,300,438) is indicated in red.

***-5kb hTERT* region II** Chr5: 1,300,062-1,300,758---(GRCh37/hg19)

*CCTGGAGAGAGGAGTCTGAG*CCTGGCTTAATAACAAACTGGGATGTGGCT<u>GGGGG**CG**^{1}GACA</u>G**CG**^{2}A**CG**^{3}G**CG**^{4}GGATTCAAAGACTTAATTC
Sp1

CATGAGTAAATTCAACCTTTCCACATC**CG**^{5}AATGGATTTGGATTTTATCTTAATATTTTCTTAAATTTCATCAAATAACATTCAGGAGTGCAGAAATC

CAAAGG**CG**^{6}TA<u>AAACAGGAACTGAG</u>CTATGTTTGCCAAGGTCCAAGGACTTAATAACCATGTTCAGAGGGATTTTT**CG**^{7}CCCTAAGTACTTTTTA
GABPA

TTGGTTTTCAT<u>AAGGTGGCTTAGGGTGCA</u>AGGGAAAGTACA**CG**^{8}AGGAGAGGGCTGGG**CG**^{9}GCAGGGCTATGAGCA**CG**^{10}GCAGGGCCAC
RAR-RXR

CG^{11}GGGAGAGAGTCCC**CG**^{12}GCCTGGGAGGCTGACAGCAGGACCACTGAC**CG**^{13}TCCTCCCTGGGAGCTGCCACATTGGGCAA**CG**^{14}**CG**^{15}AA

GG**CG**^{16}GCCA**CG**^{17}CTG**CG**^{18}TGTGACTCAGGACCCCATA<u>C**CG**^{19}GCTTCCTGGGCCCACCCAC</u>ACTA<u>ACCCAGGAAGTCA**C**</u>**G**^{20}GAGCTCTGAAC
GABPA Sp1 GABPA

C**CG**^{21}TGGAAA**CG**^{22}AACATGACCCTTGCCTGCCTGCTTCCCTGGGTGGGTCAAGGGTAATGAAGTGGTGTGCAGGAAATGGCCATGTAAATTA

CA**CG**^{23}ACTCTGCTGATGGGGAC**CG**^{24}TTCCTTCCATCAT*TATTCATCTTCACCCCCAAGGATTGAATGA*

Supplementary Figure S3: **Assessment of mutations/SNP in hTERT promoter (region I) in the cell lines studied**. The upper part of the figure is a schematic representation of the *hTERT* gene promoter (region I) and the distal regulatory region (region II). The scale of the diagram is given above the scheme. Exon 1 and part of exon 2 are represented. The locations of regions I and II are indicated. The inset in the middle part of the figure represents a zoom of the promoter region indicating the classical mutations/SNP described. In the lower part of the figure, the table depicted the presence or the absence (-) of the mutations and the SNP mentioned in the middle part.

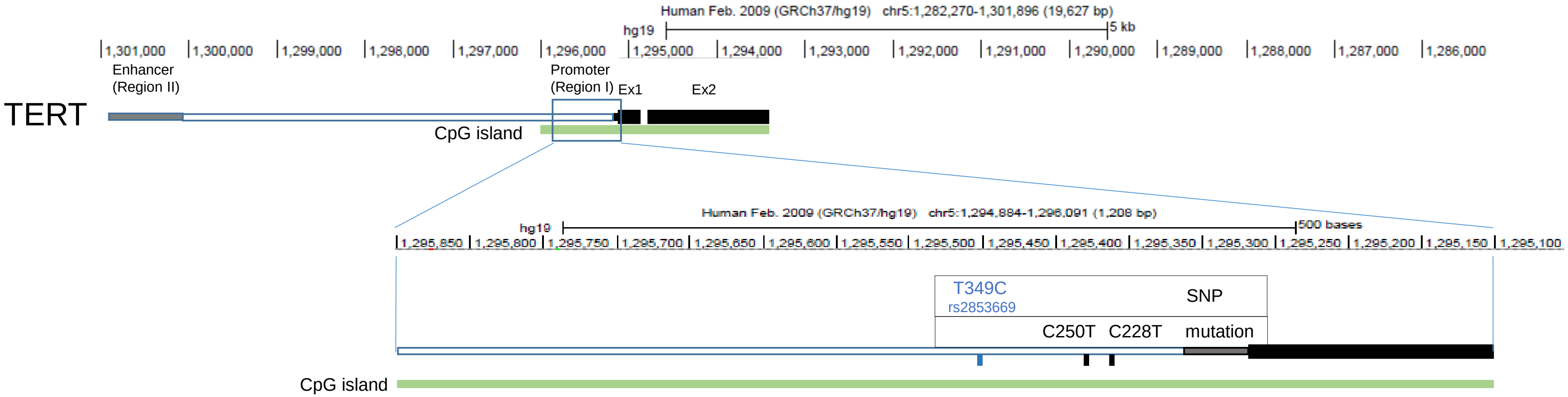

reference		T/T		C/C	C/C
ZR75.1 (luminal, **ER+**)		**C/C**		-	-
BT-474 (luminal, **Her2+**)		**T/C**		-	-
SKBr3 (luminal, **Her2+**)		-		-	-
MDA-MB-361 (**Her2+**)		-		-	-
MDA-MB-453 (basal A, **TN**)		-		-	-
MDA-MB-231 (basal B, **TN**)		-		-	**T/T**
SUM-185 PE (luminal, **TN**)		**T/C**		-	-
SUM-159 PT (basal B, **TN**)		**C/C**		-	**T/T**
MCF7 (luminal, **ER+**)		-		-	-
T47D (luminal, **ER+**)		**C/C**		-	-

Supplementary Figure S4: Assessment of mutations/SNP in the -5kb distal region of *hTERT* (region II) in the cell lines studied. The upper part of the figure is a schematic representation of the *hTERT* gene promoter (region I) and the distal regulatory region (region II). The scale of the diagram is given above the scheme. Exon 1 and part of exon 2 are represented. The locations of regions I and II are indicated. The inset in the middle part of the figure represents a zoom of the distal region indicating the previously described SNP. In the lower part of the figure, the table depicted the presence or the absence (-) of the SNP mentioned in the middle part. A 6bp deletion delCTCAGC is found, the genotype being either homozygous or heterozygous.

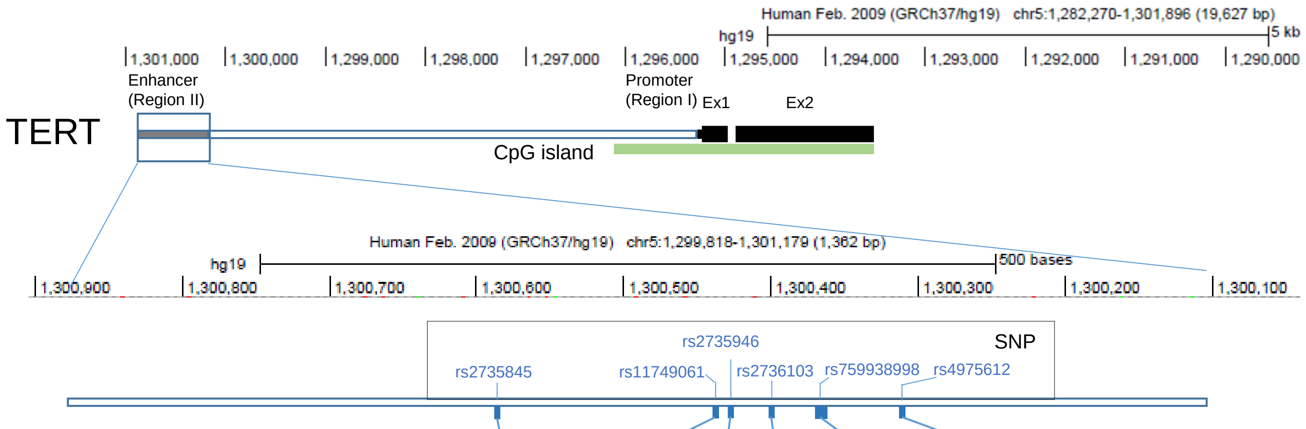

Reference	G/G		C/C	A/A	A/A	CTCAGC/ CTCAGC	C/C
ZR75.1 (luminal, **ER+**)	**C/G**		**C/T**	**C/C**	**G/G**	**delCTCAGC /delCTCAGC**	-
BT-474 (luminal, **Her2+**)	-		-	**C/C**	**A/G**	**- /delCTCAGC**	**A/C**
SKBr3 (luminal, **Her2+**)	**C/G**		**C/T**	**C/C**	**G/G**	**delCTCAGC /delCTCAGC**	-
MDA-MB-361 (luminal, **Her2+**)	-		**C/T**	**C/C**	**G/G**	**- /delCTCAGC**	**A/C**
MDA-MB-453 (basal A, **TN**)	-		-	-	-	**- /delCTCAGC**	-
MDA-MB-231 (basal B, **TN**)	-		**C/T**	**C/C**	**G/G**	**delCTCAGC /delCTCAGC**	-
SUM-185 PE (luminal, **TN**)	**C/G**		-	**C/C**	-	**- /delCTCAGC**	-
SUM-159 PT (basal B, **TN**)	-		-	**C/C**	**G/G**	**delCTCAGC /delCTCAGC**	-
MCF7 (luminal, **ER+**)	-		-	**C/A**	-	**delCTCAGC /delCTCAGC**	-
T47D (luminal, **ER+**)	-		-	**C/A**	-	**delCTCAGC /delCTCAGC**	-
